# Morphological and Genetic Clonal Diversity within the ‘Greco Bianco’ Grapevine (*Vitis vinifera* L.) Variety

**DOI:** 10.3390/plants12030515

**Published:** 2023-01-23

**Authors:** Clizia Villano, Giandomenico Corrado, Boris Basile, Ermanno Di Serio, Alessandro Mataffo, Elvira Ferrara, Riccardo Aversano

**Affiliations:** 1 Department of Agricultural Science, University of Naples Federico II, 80055 Portici, Italy; 2 Department of Environmental, Biological and Pharmaceutical Sciences and Technologies, University of Campania “Luigi Vanvitelli”, 81100 Caserta, Italy

**Keywords:** *Vitis vinifera*, ampelometry, ampelography, microsatellite, retrotransposon polymorphisms

## Abstract

Grapevine (*Vitis vinifera* L.) has been propagated vegetatively for hundreds of years. Therefore, plants tend to accumulate somatic mutations that can result in an intra-varietal diversity capable of generating distinct clones. Although it is common that winemakers request specific clones or selections for planting new vineyards, relatively limited information is available on the extent, degree, and morphological impact of the clonal diversity in traditional, highly valued grapevine varieties within production areas protected by geographical denomination of origin. Here, we present a morphological and genetic investigation of the intra-varietal diversity in ‘Greco Bianco’, the grapevine variety used to produce the DOCG and PDO “Greco di Tufo” wine. Seventeen clones from different farms (all within the allowed production area) were phenotypically characterized using ampelographic and ampelometric traits. The clones were also genotyped with Simple Sequence Repeats (SSR) and retrotransposon-based DNA markers (REMAP). The morphological analysis indicated a uniformity in the qualitatively scored traits, and a limited variability for the quantitative traits of the bunch and of the berry composition. The molecular markers also depicted variability among clones, which was more evident with the use of REMAPs. The comparison of the discriminatory information of the three analyses indicated that they provided different estimates of the level of diversity. The evaluation described herein of the clonal variability has implications for the management and protection of clonal selections in ‘Greco Bianco’ and prompts for further multidisciplinary investigations on its possible role in winemaking.

## 1. Introduction

Cultivated grapevine, *Vitis vinifera* L., is one of the most widely grown fruit crops in the world [1]. Italy ranks first in the EU as a wine producer, with 44.500 million of liters of wine produced annually. This is a consequence of the gastronomy, traditions, and pedo-climatic conditions, but also of a rich and heterogeneous cultivated germplasm. Currently, 2072 cultivars are registered in the Italian Catalogue of Grapevine Varieties (http://catalogoviti.politicheagricole.it; accessed on the 1st of December 2022). This number is probably an underestimation because the real consistency of the varieties is difficult to establish and could be higher. In the cultivated grapevine germplasm, as in other clonally propagated fruit trees, somatic mutations add genetic variability within a variety (the so-called inter-varietal diversity) [2]. For instance, bud sports are usually agamically propagated by growers and are often considered and labeled as clones. Therefore, traditional varieties should be viewed as a group of clones with genotypic, morphological, physiological, and stress-resistance traits that may not necessarily be identical to those of the original mother plant [3,4,5].

The assessment of grape diversity is an essential component of germplasm characterization and conservation, which in turn are necessary to sustain and improve crop production. Morphological traits of the fruits have often been used for the characterization of fruit trees because of their obvious economic importance and discriminating power [6]. Specifically for grape, morphological traits combined with multivariate statistical methods have been long used to assess the genetic variation and relationships among genotypes and cultivars [7,8,9,10,11]. In the last decades, grapevine discrimination methods have taken advantage of the increasing economic and technical affordability of DNA-based techniques. These can also overcome the limitations of phenotypic-based diversity analysis [12]. Among them, microsatellites (also known as Simple Sequence Repeats—SSRs) have emerged as highly versatile and informative markers for plants because they are codominant, multiallelic, and amenable to multiplexing and automatic sizing [13]. Briefly, SSR typing is based on the amplification of short, tandemly repeated sequences by using locus-specific flanking primers. For example, SSRs have been applied in studies of Italian grapes belonging to various regions [14,15,16,17,18,19,20,21,22]. These studies estimated genetic diversity in each collection and demonstrated the usefulness of these markers in discriminating varieties. Retrotransposon-based markers represent another class of anonymous and highly informative DNA markers used in plants. Briefly, retrotransposons (RTs) are dynamic and highly dispersed DNA elements, hundreds to thousands of nucleotides long, and able to translocate and change their genomic location. The various retrotransposon-based marker systems that have been developed exploit size polymorphisms (RTs cause large insertions by their transpositional activity) and the presence of conserved domains, which allow the design of specific PCR primers. For instance, in the REtrotransposon–Microsatellite Amplified Polymorphism (REMAP) system, the amplification is carried out with a primer matching the conserved end-sequence of a long terminal repeat (LTR) RT, and a primer binding to the proximity of the tandemly repeated and highly variable core sequence of an SSR locus [23]. REMAPs are cheap, easy to perform and highly polymorphic [24]. In grapevine, three retro-elements have been identified, namely Vine-1, Tvv1, and Gret1 (the only one fully sequenced) [5,25,26]. The latter is associated with mutations causing most white-fruited *V. vinifera* genotypes due to its insertion into the promoter of *VvMybA1*, which codes for a transcription factor controlling the final step in anthocyanin biosynthesis during ripening [27].

Among the rich Italian grapevine germplasm, ‘Greco Bianco’ (hereafter ‘Greco’) is a white grape variety from the Campania region used to produce the “Greco di Tufo”, an Italian wine known for its pleasant, intense, elegant, and distinctive aroma. The “Greco di Tufo” benefits from the Italian quality label “Denomination of Controlled and Guaranteed Origin” (DOCG) (DM 18.07.2003 G.U. 180, 05-08-2003), and it is also protected in the EU and the UK by the Protected Designation of Origin (PDO) geographical indication EU label (PDO-IT-A0236). It is believed that this wine has an ancient origin. One of the earliest pieces of evidence of a ‘Greco’ variety in the scientific ampelographic literature is probably the “Ampélographie (Traité général de viticulture)”, edited by Viala and Vermorel in the first decade of the last century [28]. The authors stated the hypothesis that the “Greco Bianco”, which is cultivated in the Avellino Province, is probably the same variety of the Aminaea Gemella described by ancient authors. Pliny the Elder (AD 23–79) in his *Naturalis Historia* reported the cultivation, which occurred on Mount Vesuvius and on the hills around Sorrento of Aminaea. The grapevines he described were characterized by twin bunches (*gemella*), which is a typical morphological trait of the “Greco Bianco” cultivated in modern times. From those areas, the cultivar later spread to other areas of the Campania region. Currently, the DOCG production is limited to a few towns in the Irpinia area (Avellino province), with a total area of 900 ha dedicated to viticulture (https://tinyurl.com/2p93eyyf; accessed on 1 December 2022). The wine produced in this area took the current name “Greco di Tufo”, which is probably from the tuff rock widely present in the subsoil. Interestingly, the tuff is also typical of the areas around Mount Vesuvius, as it is a common effusive magmatic rock.

The long history of cultivation of the ‘Greco’ variety suggests complex and diverse evolutionary traces following agamic propagation by farmers. Their extent and relevance at the phenotypic level is largely unknown. In this work, we examined a representative selection of varieties used by farmers to produce the DOGC “Greco di Tufo” in a long-term effort to understand the possible adaptive potential of clonal diversity in “Greco di Tufo” production and vinification. Specifically, this study aimed to analyze and quantify the intra-varietal diversity of the ‘Greco’ using DNA analysis based on two marker systems (microsatellite and REMAP markers) and a morphological characterization mainly focusing on pomological traits. Moreover, in a first attempt to understand the relevance of the observed diversity, we correlated genetic and morphological distances. The gathered information helps design germplasm maintenance strategies and, ultimately, would guide selection and breeding.

## 2. Results

### 2.1. Diversity in Morphological and Berry Composition Traits

The 17 clones did not differ in terms of the ten qualitative morphological traits analyzed in this study. In summary, they all were characterized by (a) shoots with two consecutive tendrils; (b) mature leaves having three lobes, a cordate/pentagonal blade shape, teeth straight on both sides, a U-shaped base of both the petiole and the upper lateral sinuses; (c) flowers with fully developed stamen and gynoecium; (d) conical bunch shape; (e) one wing in the primary bunch; and (f) a broad-ellipsoid berry shape.

Bud potential fertility index (PFI) did not differ among the 17 clones (with an average PFI of 1.2 bunches per shoot), whereas, at harvest, bunch morphology varied (Table 1). Large variability was found among the clones in the components of bunch compactness. The number of berries per bunch, the total rachis length, and the bunch compactness index ranged, respectively, between 77 (G16) and 157 berries per bunch (G14), between 10.6 (G12) and 21.1 cm (G1), and between 4.77 (G5) and 9.90 berries/cm (G11), respectively. Clones G11 and G14 had the most compact bunches mainly because of the high number of berries per bunch (133 and 157 berries per bunch, respectively), which is also associated, in the case of G11, with a short rachis length (14 cm). However, G1, G5, and G16 had the loosest bunches (an average IC of 4.92 berries per bunch), mainly because of a long rachis (G1 and G16) or a low number of berries per bunch (G5). The other clones had bunches with intermediate compactness (Table 1).

Berry composition at harvest differed significantly between clones (Table 2). G1 and G17 had the highest juice TSS (around 24.3 °Brix) and intermediate TA values (10.4 and 9.6 g/L tartaric acid, respectively), whereas G9 berries were characterized by relatively low TSS (20.2 °Brix) and TA (7.95 g/L tartaric acid). Juice TA at harvest was lowest in G12 (7.2 g/L tartaric acid). The other clones had intermediate TSS (between 21.0 and 23.6 °Brix) and TA (between 8.55 and 12 g/L tartaric acid). Juice pH at harvest also significantly varied between clones ranging between 2.96 (G2) and 3.21 (G6 and G12). G12 berries had the highest TSS/TA ratio (3.16), followed by G7 with TSS/TA values of 2.62. The lowest values of the TSS/TA ratio were measured in G2 and G3 (Table 2). The other clones had TSS/TA values between 1.87 (G14) and 2.55 (G15).

**Figure 1:**
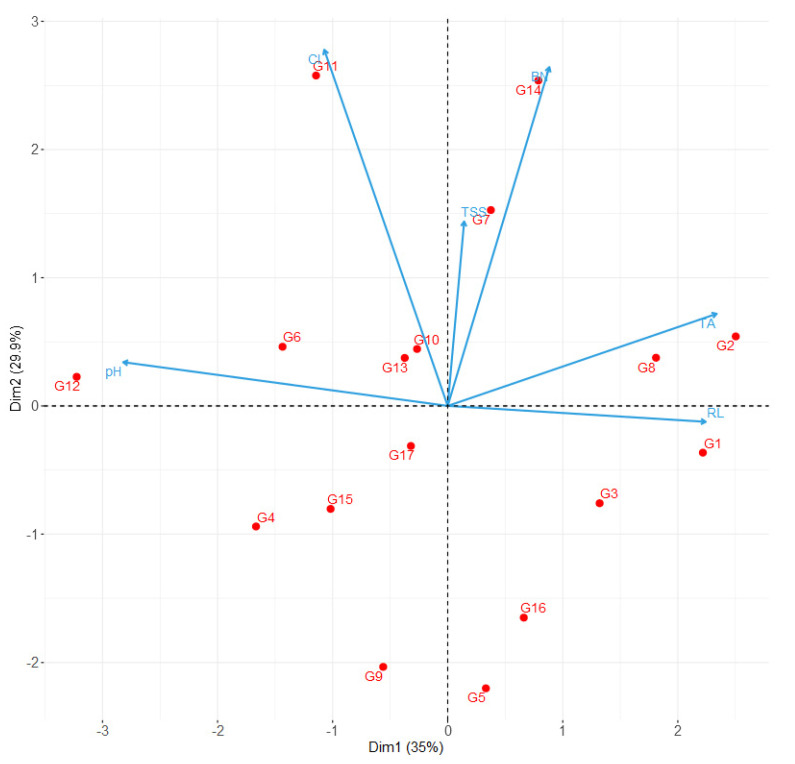
Bi-plot of the 17 ‘Greco’ clones (from G1 to G17) for the first principal components extracted by the PCA. The vectors indicate the original variable loadings. BN—number of berries per bunch; RL—Rachis length; CI—bunch compactness index; TSS—berry juice total soluble solids; pH—berry juice pH; TA—berry juice titratable acidity.

For multivariate exploratory analysis, we removed from the initial 18 parameters those that were not statistically different among clones, namely the ten qualitative traits and PFI. The PCA extracted a total of six principal components, with the first three having eigenvalues higher than 1 and explaining together 82.6% of the total variance (Table 3). The first principal component (Dim. 1) was positively correlated with rachis length and TA, and negatively with pH (Table 3 and Figure 1). The second principal component (Dim. 2) correlated positively with the number of berries per bunch and bunch compactness index. Dim. 3 correlated positively with rachis length (Table S1 and Figure 1).

The bi-dimensional spaces delineated combining the first two principal components (Figure 1) separated the 17 clones well. Specifically, they were uniformly scattered, as indicated, for instance, by the almost equal number of clones in each quadrant, and many were distant from the plot origin. Moreover, clusters or similar clones were not evident even when a priori knowledge and possible groups related to the geographical locations were taken into account. Finally, the PCA bi-plot indicated that the patterns of morphological variance depicted by the PCs may have different, un-orthogonally correlated causes given the uniform distribution and the correlation of the variable loadings.

### 2.2. Genetic Diversity

#### 2.2.1. Microsatellite Analysis

The DNA of the 17 clones under investigation was analyzed with ten highly polymorphic SSR loci described in the literature (Table S1). All loci were polymorphic in our population (presenting more than one allele), and co-dominance was confirmed by the presence of a maximum of two alleles per clone. Main genetic indexes are reported in Table 4.

We obtained 29 alleles in total, with an average of 2.9 ± 0.31 (mean ± s.e.) alleles per locus, and 14 different multilocus genotypes. The size of the alleles ranged from 112 bp (VRZAG29) to a maximum of 320 bp (VVIp60) and was consistent with the literature. As expected for clonally propagated crops, the observed heterozygosity was very high (0.87 ± 0.10), and only two loci had homozygous genotypes; although the Ho was high (i.e., above 50%) for the VVMD7 locus, the other locus (VVS5) was almost fixed. The Expected heterozygosity (He), Fixation index (F), and Shannon’s Index were, on average, 0.50 ± 0.03, −0.68 ± 0.18, and 0.78 ± 0.05, respectively. The He was greater than or equal to 0.5 in all markers except VVS5. Values were always below 0.6, suggesting that the high level of the Ho is not due to a highly uniform distribution of the alleles. This was confirmed by the estimation of the Evenness, a measure related to the ratio between the more abundant and the rarer genotypes, whose high values were overall consistent with clonal diversity. Consequently, the F value was negative for all markers except VVS5 (0.848). The Shannon’s index was close to 1.0 in VVMD7 and lower than 0.8 in the other markers.

It was not possible to find a reference ‘Greco’ genotype considering all the markers mainly because of the variability at VVMD7. Specifically, for the biallelic VRZAG21, VVS4, VRZAG29, and VVLN16 loci, we detected only one heterozygote profile in the 17 clones. A highly predominant allelic combination (i.e., 94%) was found in VVS2 and VVIC05, having four and two alleles, respectively. For VVLH54, VVS5, and VVLP60, a common heterozygote profile was present in at least 75% of the genotypes. Six genotypes were present at the tetra allelic VVMD7 locus yet, among them, two were predominant, with a 29% frequency each. Overall, the genotypic analysis is consistent with a highly heterozygote population of clonal origin. The analysis of the VVMD7 locus suggests the presence of two main groups which may have derived from an early differentiation. Finally, the SSR analysis indicated intra-clonal variability at six of the ten employed SSRs.

**Figure 2:**
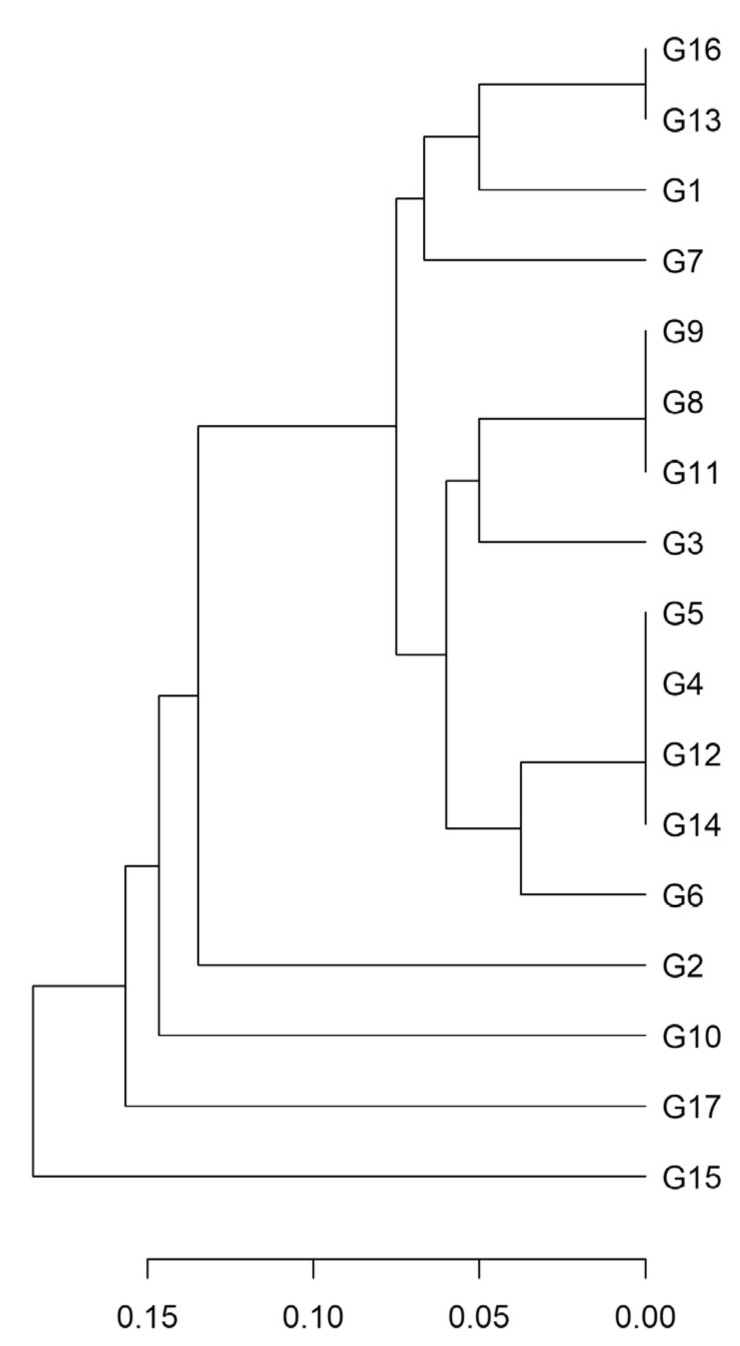
Dendrogram of the ‘Greco’ clones (G) based on their SSR profile. The dendrogram was built with the UPGMA algorithm using Provesti’s genetic distance (reported in the scale bar on the bottom).

To illustrate the genetic resemblance among clones, we built a dendrogram using Provesti’s genetic distance and the UPGMA algorithm (Figure 2). The correlation between the distance and the cophenetic matrices was high (r = 0.85) and significant (*p* ≤ 0.001; Mantel test), indicating high goodness of fit. The average distance was very low (0.10 ± 0.01), and three groups of clones were indistinguishable. Specifically, the clones G16 and G13, G8, G9, and G11, and G4, G5, G12, and G14 had an identical SSR profile.

#### 2.2.2. REMAP Analysis

**Figure 3:**
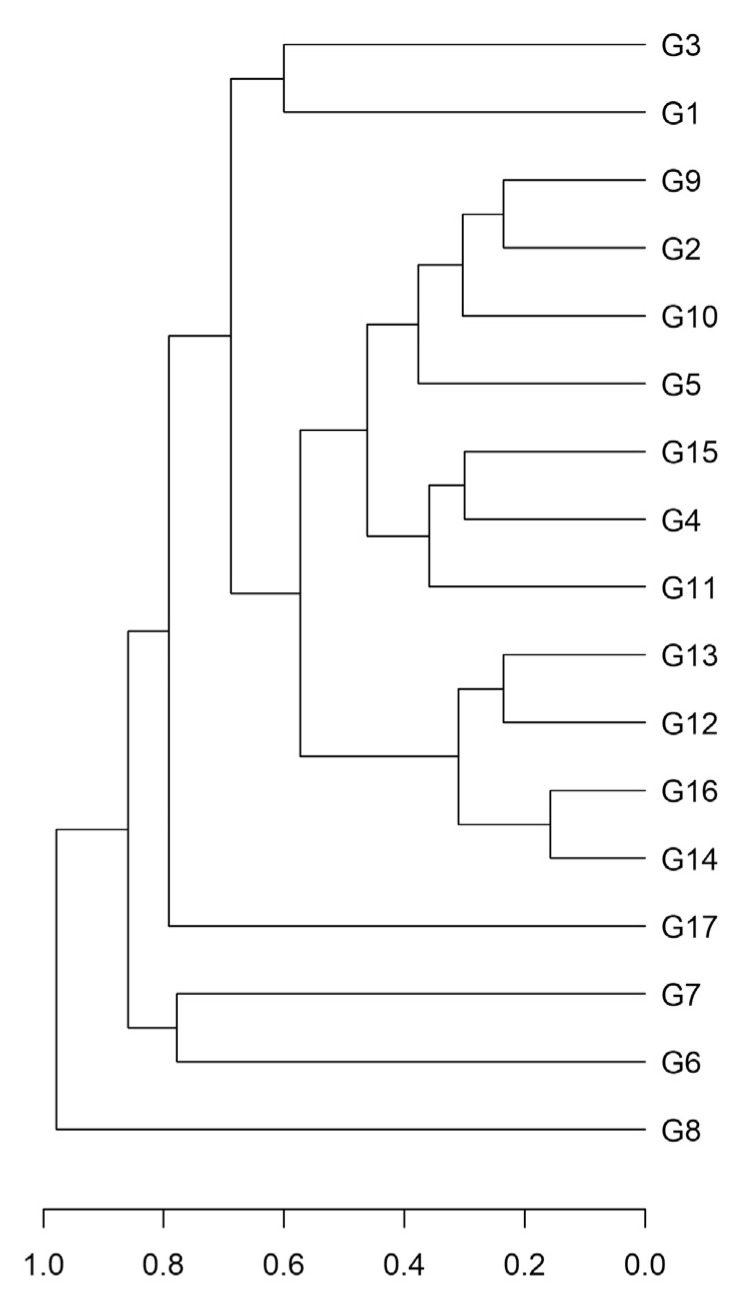
Dendrogram of the ‘Greco’ clones (G) based on the REMAP profile. The dendrogram was built with the UPGMA algorithm using the Jaccard genetic distance (reported in the scale bar on the bottom).

To complement and extend the analysis of the genetic diversity among clones, we also used the highly polymorphic REMAP marker system based on the Gret1 LTR elements. We obtained 26 easily scorable polymorphic fragments in our population, with an average of 11.77 bands per clone. All ‘Greco’ clones had a unique molecular profile. The relationship among clones is illustrated by the hierarchical cluster obtained using the Jaccard distance and the UPGMA algorithm (Figure 3). The linear correlation between the distance and the cophenetic distance matrices was very high (0.93) and significant (*p* ≤ 0.001; Mantel test), indicating very high goodness of fit of the dendrogram with the dissimilarity matrix on which it was based.

### 2.3. Relationships between the Classification Obtained with the Different Marker Systems

To test for agreement between the hierarchical classification obtained with morphological, SSR, and REMAP data, we compared matrices using the matrix correlation coefficient as their index of concordance (by analyzing corresponding values in two half-matrices, diagonal excluded) [29]. Moreover, a comparison of cophenetic matrices was made possible using matrices obtained with the same clustering algorithm. To this aim, we first calculated a distance matrix for the quantitative data using the Gower’s index on scaled (Z-score) data and the related cophenetic matrix based on the UPGMA algorithm. The goodness of fit between the matrices relative to the quantitative morphological traits was not high 0.71 (*p* ≤ 0.001; Mantel test). Afterwards, we performed the concordance test on distance and cophenetic matrices (Table 5).

The pairwise correlation between the two DNA marker systems and the quantitative traits was always not significant, both in terms of described differences among clones and at the hierarchical classification level, indicating that the different systems depicted different sources of variability.

## 3. Discussion

Knowledge of the differences between plants belonging to the same grape variety is useful to manage germplasm, ensure homogeneity of new vineyards, and select superior clones even when considering the limitation of genetic exchange-based breeding approaches in grapevine. In this work, we evaluated the on-farm intra-varietal diversity of the traditional ‘Greco’ grapevine in the PDO area. Previous studies investigated the clonal diversity present in plants collected in ample areas [30,31,32]. Although the long history of cultivation and farmer-to-farmer exchange (as an alternative to the use of certified material) may allow for a more diverse population [33], it is also expected that the propagation of a relatively reduced number of mother plants, along with the more recent pressure to comply with production and processing rules, will restrain the clonal diversity in the PDO areas.

For the estimation of phenotypic variability, leaf and fruit descriptors have been widely used for both grapevine cultivars and clones [34,35,36,37]. Clones typically need multivariate biometric analysis to be distinguished [38]. Our work indicated heterogeneity in some morphological traits, such as rachis length and bunch compactness, which are under strong genetic control [39]. The multivariate data analysis did not reveal highly correlated influential variables, whereas a rather uniform distribution of the plants was observed, all of which suggest that separate, independent sources are responsible for the pattern of morphological variability among the clones. Although the qualitative traits were not polymorphic, differences were detected not only for the bunch but also for the berry composition. This is consistent with previous studies that indicate the high discriminating power of berry composition in distinguishing grape genotypes [7,9]. Although the variation of this trait is often limited (considering the CV value), it remains to be tested if the clonal diversity may be associated with different specific compounds and, ultimately, wine flavors [40].

We also investigated the genetic diversity of two DNA-based markers, SSR and REMAP. The diverse nature of these markers revealed a different level of variability within clones. For instance, SSRs were unable to distinguish all the genotypes, consistent with previous knowledge indicating that this marker does not often show allelic variation among grape clones [41,42]. The analysis of the co-dominant SSRs revealed a high genetic similarity, fixed heterozygosity at several loci, and the frequent presence of predominant locus-specific profiles. Taken together, these can be considered evidence of the common origin of the material. Therefore, it is possible to propose for further evaluation that the ‘Greco’ of the PDO area is of monoclonal origin [32,38]. This is also supported by the fact that divergent genotypes usually differed for very few loci. Moreover, the analysis of the most variable SSR locus (VVMD7) implied the possibility of two main groups of clones. On the other hand, REMAP markers separated all the genotypes under investigation. Considering the lack of genetic recombination and the abundance of transposable elements in the grape genome, insertion polymorphisms due to the activity of mobile elements are considered the largest source of clonal diversity in cultivated grapevine [43,44]. For instance, retrotransposon-based markers can identify polymorphisms within a single grapevine clone even when SSRs cannot resolve the existing genetic diversity [25,45,46]. Although the REMAP marker system proved to be a useful tool for fingerprinting individual plants, its traditional disadvantages are the lack of co-dominance and association with phenotypic traits. Overall, our work confirmed the usefulness of using different DNA typing methods for the analysis of the diversity among clones [30] and that their combination is probably necessary for a better understanding of their evolutionary history.

From a molecular perspective, the polymorphism of SSRs and REMAPs originate from independent mechanisms, but the agamic propagation of the germplasm should not allow different evolutionary trajectories, making possible a potential correlation. The comparison of the classification provided by the two DNA marker systems indicated that they provided different information, also considering the analysis of the cophenetic values (i.e., the similarity levels at which two entities group together during clustering). Other works based on the diversity among grapevine and varieties indicated that the cophenetic matrix of REMAP significantly correlated with that of the Inter-Single Sequence Repeats (ISSR) markers, but not with that of SSR, AFLP, SSAP, or IRAP [25]. Likewise, also the relationship between the morphological and each of the two molecular classifications was very low and not significant. The literature on agamically propagated fruit trees reports usually low or non-significant correlations between DNA-based and morphological classifications [22,47,48,49]. This is typically explained by the fact that the employed marker systems sampled a different kind of mostly uncorrelated diversity. It is very likely that the two neutral DNA marker systems principally tested a non-adaptive diversity. Nonetheless, it is also necessary to add that interesting agronomic features are fixed in grapevine through clonal selection and agamic propagation, thus ensuring the concurrent conservation of both adaptive and neutral genetic diversity. An implication that should be tested analyzing an ample panel of grapevine varieties is that it will be unlikely to infer the presence of phenotypic differences among grape clones according to the of resemblance provided by anonymous and/or neutral DNA markers. Therefore, the classification, distinction, and descent of the clones as well as the identification of a reference genotype for the ‘Greco’ variety should be based on a multidisciplinary integration of different tools.

In conclusion, this work assessed the clonal diversity of a traditional grapevine. The data indicated a limited phenotypic variability and supported a monoclonal origin of the ‘Greco’ population present the PDO area. Although our aim did not include the identification of clone-specific variants and demographic assessments (e.g., parentage reconstruction), the SSR and REMAP analysis suggested a possible division in two groups and the presence of region-specific genomic differences given the highly uncorrelated classifications. Further validations, which should also include material outside the PDO area, will be able to identify a reference molecular profile for clone authenticity and for the future identification of adaptive genetic variation.

## 4. Materials and Methods

### 4.1. Plant Material and Experimental Site

The grapevine germplasm under investigation included 17 clones of *Vitis vinifera* cv. ‘Greco Bianco’ sampled in different areas of Tufo village (Avellino), Italy (Table S1). During the experiment, air temperature was measured hourly at a weather station located within 2 km from the experimental sites (Figure S1).

### 4.2. Morphological and Berry Composition Analysis

Ten qualitative and three quantitative morphological traits were assessed on the vines of the 17 selected clones in 2019. The qualitative morphological descriptors were categorically scored according to the indications of the OIV (available at https://www.oiv.int/public/medias/2274/code-2e-edition-finale.pdf, accessed on 1 December 2022): number of consecutive tendrils on the shoots (descriptor identification code: OIV-016), blade shape in mature leaves (OIV-067), number of lobes in mature leaves (OIV-068), teeth shape in mature leaves (OIV-76), shape of base of petiole sinus in mature leaves (OIV-080), shape of the base of upper lateral sinuses in mature leaves (OIV-083-1), morphological development of sexual organs in flowers (OIV-151), bunch shape (OIV-208), number of wings in the primary bunch (OIV-209), and berry shape (OIV-223). The quantitative traits measured in this study were the number of berries per bunch, the total rachis length (main axis + wing axes), and the compactness index (calculated as the number of berries per bunch to total rachis length ratio). These parameters were measured at harvest on five bunches per clone.

Right after fruit set, the number of bunches located on eight shoots per vine was counted separately and was used to calculate the bud fertility potential index (mean number of bunches per shoot). Grape harvest was carried out when total soluble solids (TSS) in berry juice reached around 21 °Brix. TSS was measured on the extracted juice with digital refractometer (HI96811, Hanna Instruments, TX, USA). For clones G1, G2, G3, G4, G5, G6, G8, G10, G11, G13, G14, and G16, grapes were harvested on October 2 whole for G7, G9, G12, G15, and G17 6 days later (8 October). At harvest, three 20-berry samples were collected per vine and transported to the laboratory for measuring total soluble solids (TSS), pH, and titratable acidity (TA) of berry juice. TSS was measured as previously described, whereas juice TA was measured by titration with a 0.1 M NaOH solution until reaching a pH value of 8.2 and expressed as g/L tartaric acid. The pH was monitored continuously with a pH-meter (GLP 21; Crison, Alella, Barcelona, Spain). The TSS/A ratio was calculated for each sample.

The significance of the differences among clones in the quantitative morphological traits was assessed with one-way ANOVA using the Duncan as a post-hoc test for mean separation (*p* ≤ 0.05). Moreover, differences between clones in these traits were studied with a Principal Component Analysis (PCA) using, as input, seven original variables: no berries per bunch, rachis length, bunch compactness index, berry juice TTS, pH, TA, and the TSS/TA ratio. Statistical analyses and biplot representation were achieved using R 4.2.

### 4.3. Microsatellite Analysis

For DNA analysis, six young healthy leaves per plant (in three sampled replicates per clone) were harvested and immediately frozen with liquid nitrogen. Total genomic DNA was extracted using the Plant DNeasy Maxi Kit (Qiagen, Milan, Italy) as per the manufacturer’s procedure. Microsatellite analyses were carried out with ten nuclear markers (Table S2) selected from different sources [42,50,51,52]. The amplification profile was as reported [21]. PCR reactions were performed in a 20 μL volume containing 1 × reaction buffer with 1.6 mM MgCl_2_, 0.2 mM of each dNTP, 30 pM FAM-labelled forward SSR primer, 30 pM reverse SSR primer (Table S2), 1 unit of Taq polymerase (Promega, Milan, Italy), and 30 ng of genomic DNA. After agarose gel electrophoresis to verify the correct amplification and estimate product size, amplicons were separated with the ABI PRISM 3130 DNA Analyzer system (Applied Biosystems, Milan, Italy). Size calibration was performed with the DNA marker size ladder GenScan 500 ROX dye Size Standard (Applied Biosystems). Binning and sizing of the SSR alleles were performed using the Peak Scanner software v 2.0 (Applied Biosystems). Experiments were carried in triplicate per clone.

### 4.4. REMAP Analysis

For REMAP markers amplification, three primers were chosen: one designed on the Gret1 LTR region 3′-LTR (5′- GCATTTAGAAGGATTTAGCTT-3′) and two anchored primers designed on microsatellite repeats, Microsat-GA ((GA)_9_C) and Microsat-CT ((CT)_9_G). We set up two REMAP markers that combine the LTR primer with the microsatellite primers as described (Pereira et al., 2012). PCR reactions were performed in a 20 μL reaction mixture containing 20 μg of genomic DNA template, as previously described [24]. PCR products were separated via 2% agarose gel electrophoresis at 50 V for 30 min, 70V for 30 min, and then 100 V for 3 h in 1 × TAE buffer. The bands were detected using the GelRed Nucleic Acid Gel Stain (Biotium, Hayward, CA, USA). For the image acquisition and the identification of band size, the Quantity One 1-D Analysis Software (Bio-Rad Laboratories, Hercules, CA, USA) was used. Experiments were carried in triplicate per clone.

### 4.5. Molecular Data Analysis

The GenAlex 6.5 software [53] was used to obtain the main indices of genetic diversity based on the SSR profiles, namely the number of alleles (Na); the allelic size range in bp; the observed heterozygosity (Ho); the expected heterozygosity (He): 1 − Σp_i_^2^; the Shannon’s index of diversity (I): −1 × Σ(p_i_  ×  ln(p_i_)); the Wright’s fixation index (F): (He − Ho)/He, where for each locus, p_i_ is the frequency of the i-th allele, Σp_i_^2^ is the sum of the squared population allele frequencies. The Poppr library [54] was used to calculate the Evenness (E) as [(1/λ) − 1]/[(e^H^) − 1], where 1/λ is Stoddart and Taylor’s index, and H is the Shannon diversity.

Genetic distances were calculated using the Prevosti distance [55], which ranges from 0 to 1. REMAP fragments, scored as binary markers and were used to calculate the Jaccard distance [56], which ranges from 0 to 1. Cluster analysis was performed using the unweighted pair group method with arithmetic mean (UPGMA) algorithm [56].

### 4.6. Concordance Test between Marker Systems

For each marker type (i.e., morphological data, SSR and REMAP), ordered parallel triangular matrices were obtained using the matsbyname R package. From independent data, pairwise comparison of distance matrices and the cophenetic matrices obtained with the same algorithm (UPGMA) was performed in R 4.2 with the Mantel test (9999 permutations) using the ADE4 package [57].

## Figures and Tables

**Table 1:** Bud potential fertility and bunch morphological traits. In each column, different statistical letters indicate statistically significant groups according to a one-way ANOVA followed by Duncan post-hoc test (*p* ≤ 0.05). The Coefficient of Variation (C.V.) among clones was reported for each measured parameter.

Clone	Bud Potential Fertility Index	Components of Bunch Compactness		
		No Berries Per Bunch	Rachis Length (cm)	CI (No Berries/ cm Rachis)
G1	1.10 ± 0.14 ^a^	108 ± 23 ^ab^	21.1 ± 2.6 ^a^	4.99 ± 0.62 ^e^
G2	1.00 ± 0.14 ^a^	114 ± 19 ^ab^	16.3 ± 0.8 ^abcd^	6.86 ± 0.85 ^bcde^
G3	1.00 ± 0.15 ^a^	86 ± 13 ^b^	14.4 ± 2.0 ^abcd^	6.33 ± 1.42 ^bcde^
G4	1.18 ± 0.18 ^a^	78 ± 13 ^b^	12.4 ± 2.0 ^cd^	6.53 ± 0.97 ^bcde^
G5	1.27 ± 0.18 ^a^	77 ± 14 ^b^	16.0 ± 1.3 ^abcd^	4.77 ± 0.79 ^e^
G6	1.19 ± 0.14 ^a^	110 ± 33 ^ab^	14.8 ± 2.7 ^abcd^	7.07 ± 1.14 ^bcde^
G7	1.33 ± 0.19 ^a^	137 ± 27 ^ab^	17.8 ± 3.5 ^abc^	7.71 ± 0.03 ^abcd^
G8	1.29 ± 0.167 ^a^	129 ± 15 ^ab^	19.9 ± 0.8 ^ab^	6.43 ± 0.55 ^bcde^
G9	1.29 ± 0.17 ^a^	91 ± 20 ^b^	17.3 ± 2.4 ^abcd^	5.15 ± 0.47 ^de^
G10	1.29 ± 0.19 ^a^	134 ± 1 ^ab^	19.3 ± 1.3 ^ab^	7.07 ± 0.45 ^bcde^
G11	1.17 ± 0.14 ^a^	133 ± 21 ^ab^	14.0 ± 2.9 ^bcd^	9.90 ± 0.77 ^a^
G12	1.20 ± 0.17 ^a^	87 ± 12 ^b^	10.6 ± 1.8 ^d^	8.28 ± 0.72 ^abc^
G13	1.12 ± 0.17 ^a^	118 ± 15 ^ab^	15.9 ± 0.8 ^abcd^	7.37 ± 0.69 ^bcde^
G14	1.13 ± 0.17 ^a^	157 ± 16 ^a^	18.0 ± 0.9 ^abc^	8.64 ± 0.50 ^ab^
G15	1.29 ± 0.167 ^a^	91 ± 14 ^b^	15.9 ± 2.1 ^abcd^	5.91 ± 0.91 ^cde^
G16	1.38 ± 0.18 ^a^	96 ± 5 ^b^	20.0 ± 1.9 ^ab^	4.98 ± 0.65 e
G17	1.19 ± 0.16 ^a^	91 ± 13 ^b^	15.9 ± 1.9 ^abcd^	5.76 ± 0.51 ^cde^
C.V. (%)	9.1	21.9	16.9	21.1

**Table 2:** Berry composition of the different clones at harvest. The harvest date for clones G7, G9, G12, G15, and G17 (8 October 2019) was six days later than the other clones (2 October 2019). In each column, different statistical letters indicate statistically significant groups according to a one-way ANOVA followed by Duncan post-hoc test (*p* ≤ 0.05). The Coefficient of Variation (C.V.) among clones was reported for each measured parameter.

Clone	TSS (°Brix)	pH	TA (g/L Tartaric Acid)	TSS/TA
G1	24.3 ± 0.3 ^a^	3.00 ± 0.06 ^cd^	10.4 ± 0.1 ^abcd^	2.35 ± 0.03 ^de^
G2	22.6 ± 0.2 ^bcd^	2.96 ± 0.04 d	14.1 ± 0.1 ^a^	1.60 ± 0.01 ^i^
G3	21.6 ± 0.2 de	3.01 ± 0.02 ^cd^	13.4 ± 2.2 ^ab^	1.62 ± 0.01 ^i^
G4	22.3 ± 0.3 ^cd^	3.15 ± 0.01 ^abc^	8.9 ± 0.1 ^cd^	2.52 ± 0.04 ^bc^
G5	21.1 ± 0.4 ^ef^	3.07 ± 0.01 ^abcd^	10.1 ± 0.4 ^abcd^	2.10 ± 0.04 ^f^
G6	22.8 ± 0.5 ^bc^	3.21 ± 0.09 ^a^	9.3 ± 2.1 ^bcd^	2.45 ± 0.06 ^cd^
G7	23.6 ± 0.2 ^ab^	3.07 ± 0.01 ^abcd^	9.0 ± 0.3 ^cd^	2.62 ± 0.02 ^b^
G8	22.2 ± 0.3 ^cd^	3.03 ± 0.04 ^bcd^	11.0 ± 0.4 ^abcd^	2.03 ± 0.03 ^fg^
G9	20.2 ± 0.4 ^f^	3.13 ± 0.04 ^abc^	8.0 ± 1.3 ^cd^	2.55 ± 0.05 ^bc^
G10	21.2 ± 0.2 ^ef^	3.16 ± 0.01 ^ab^	8.6 ± 0.1 ^cd^	2.47 ± 0.026 c
G11	23.2 ± 0.2 ^bc^	3.15 ± 0.03 ^abc^	9.9 ± 0.9 ^abcd^	2.34 ± 0.02 de
G12	22.8 ± 0.3 ^bcd^	3.21 ± 0.04 ^a^	7.2 ± 0.1 d	3.16 ± 0.046 ^a^
G13	21.1 ± 0.6 ^ef^	3.16 ± 0.07 ^ab^	10.8 ± 2.7 ^abcd^	1.95 ± 0.06 gh
G14	22.5 ± 0.1 ^cd^	3.13 ± 0.07 ^abc^	12 ± 2.4 ^abc^	1.87 ± 0.01 h
G15	22.5 ± 0.7 ^bcd^	3.17 ± 0.02 ^ab^	8.9 ± 0.1 ^cd^	2.55 ± 0.08 ^bc^
G16	21.1 ± 0.4 ^ef^	3.10 ± 0.01 ^abc^	9.3 ± 0.3 ^bcd^	2.27 ± 0.04 e
G17	24.4 ± 0.3 ^a^	3.13 ± 0.04 ^abc^	9.6 ± 0.9 ^bcd^	2.54 ± 0.03 ^bc^
C.V. (%)	5.2	2.3	18.1	17.2

**Table 3:** Eigenvalue, percent of explained variance, and correlation with the eight original variables of the six principal components (Dim.) extracted. BN—number of berries per bunch; RL—Rachis length, CI—bunch compactness index; TSS—berry juice total soluble solids; pH—berry juice pH; TA—berry juice titratable acidity; TSS/TA—TSS to TA ratio.

Principal Component	Eigenvalue	Variance Explained (%)	Correlation						
			BN	RL	CI	TSS	pH	TA	TSS/TA
Dim. 1	2.10	35.0	0.28 ^NS^	0.72 ^**^	−0.34 ^NS^	0.05 ^NS^	−0.91 ^***^	0.75 ^***^	0.28 ^NS^
Dim. 2	1.79	29.9	0.85 ^***^	−0.04 ^NS^	0.89^***^	0.46 ^NS^	0.11 ^NS^	0.23 ^NS^	0.85 ^NS^
Dim. 3	1.06	17.7	0.44 ^NS^	0.64 ^**^	−0.10 ^NS^	−0.40 ^NS^	0.30 ^NS^	−0.44 ^NS^	0.44 ^NS^
Dim. 4	0.88	14.7	−0.06 ^NS^	0.25 ^NS^	−0.24 ^NS^	0.79 ^**^	−0.01 ^NS^	−0.37 ^NS^	−0.06 ^NS^
Dim. 5	0.15	2.5	0.01 ^NS^	0.04 ^NS^	−0.12 ^NS^	0.06 ^NS^	0.28 ^NS^	0.23 ^NS^	0.01 ^NS^
Dim. 6	0.01	0.2	−0.07 ^NS^	0.06 ^NS^	0.07 ^NS^	0.01 ^NS^	0.01 ^NS^	0.01 ^NS^	−0.07 ^NS^

**, ***, and ^NS^ indicate significant differences at *p* ≤ 0.01, *p* ≤ 0.001, and not significant (*p* > 0.05) correlation.

**Table 4:** Main genetic indexes of diversity in the analyzed population of ‘Greco’ clones. Na—number of alleles; ASR—allelic size range; Ho—observed heterozygosity: He—expected heterozygosity; I—Shannon’s index of diversity; F—Wright’s fixation index; and E—evenness.

Locus	Na	ASR	Ho	He	I	F	E
VVS2	4	128–126	1.00	0.56	0.92	−0.80	0.83
VVMD7	4	244–250	0.65	0.58	1.05	−0.12	0.75
VRZAG21	2	188–200	1.00	0.50	0.69	−1.00	1.00
VVS4	2	166–172	1.00	0.50	0.69	−1.00	1.00
VRZAG29	2	112–118	1.00	0.50	0.69	−1.00	1.00
VVLN16	2	149–157	1.00	0.50	0.69	−1.00	1.00
VVLH54	3	163–167	1.00	0.53	0.82	−0.88	0.90
VVS5	4	147–167	0.06	0.28	0.60	0.78	0.47
VVIC05	2	162–166	1.00	0.50	0.69	−1.00	1.00
VVLP60	4	312–320	1.00	0.56	0.94	−0.78	0.83

**Table 5:** Pairwise comparison of molecular and morphological data. Numbers in parentheses indicate *p*-value (Mantel test). The results below (respectively, above) the diagonal indicated the correlation coefficient between the distance matrices (resp. the cophenetic value matrices).

	Quantitative	SSR	REMAP
Quantitative	-	−0.034 (0.54)	−0.074 (0.64)
SSR	−0.120 (0.76)	-	−0.084 (0.64)
REMAP	−0.150 (0.82)	0.028 (0.43)	-

## Data Availability

Data that are not already contained within the article or supplementary material will be available from the corresponding author (B.B.) upon reasonable request.

## Supplementary Materials

The following supporting information can be downloaded at: https://www.mdpi.com/article/10.3390/plants12030515/s1, Figure S1: Minimum, mean, and maximum air daily temperature (2 m); each dot represents a daily measurement; lines are smoothed conditional means surrounded by standard error of the means (grey areas). Table S1. Plant material under investigation and their location; Table S2. Microsatellite markers used for grape genotyping and their main features (linkage group, primer sequences (5′–3′), core motif, and annealing temperatures (Ta) in °C).
