# Supplementary material for: Morphological and Genetic Clonal Diversity within the ‘Greco Bianco’ Grapevine (Vitis vinifera L.) Variety
Source: Plants (Basel). 2023 Jan 23;12(3):515. doi: 10.3390/plants12030515 (PMC9921137; doi:10.3390/plants12030515)
Supplement: Supplementary file 1 [file plants-12-00515-s001.zip › Supplementary Tables.pdf]

Table S1. Plant material under investigation

Clone	Abbreviation	Year of tree planting	Training system	Longitude	Latitude
Greco 1	G1	1980	Guyot	14.83515	41.00951
Greco 2	G2	1990	Guyot	14.83644	41.00830
Greco 3	G3	1990	Guyot	14.83610	41.00777
Greco 4	G4	1990	Guyot	14.83593	41.00753
Greco 5	G5	1985	Guyot	14.84205	41.00558
Greco 6	G6	1980	Tendone	14.84208	41.00294
Greco 7	G7	1980	Guyot	14.81752	41.01826
Greco 8	G8	1975	Guyot	14.81880	41.01926
Greco 9	G9	1980	Guyot	14.81920	41.01883
Greco 10	G10	1983	Guyot	14.82028	41.02161
Greco 11	G11	1948	Tendone	14.81981	41.01899
Greco 12	G12	1985	Guyot	14.81929	41.02036
Greco 13	G13	1990	Tendone	14.82355	41.01849
Greco 14	G14	2014	Guyot	14.82308	41.01777
Greco 15	G15	1985	Guyot	14.82579	41.01852
Greco 16	G16	1980	Guyot	14.82542	41.01909
Greco 17	G17	1975	Guyot	14.82269	41.01911

Table S2. Microsatellite markers used for grape genotyping. Locus name, linkage group, primer sequences (5′-3′), core motif, and annealing temperatures (Ta) in °C.

Locus	Linkage Group	Forward primer	Reverse primer	Core motif	Ta (°C)	Reference
VrZAG21	4	tcattcactcactgcattcatcggc	ggggctactccaaagtcagttcttg	(GA)	61	Sefc et al.,1999
VrZAG29	1	ataaccaggacaaagttattcaagcc	acccaattgaccatcttttatgctg	(GA)	48	Sefc et al.,1999
VVIC05	11	aagcaagttgaagaacgtgtaagtc	ggcgaagaatcttactgagaattg	(CT)	50	Merdinoglu et al.,2005
VVIh54	13	ccgcacttgtgttgaatttcag	caaaccgtttttacaccagcag	(GA)	56	Laucou et al.,2011
VVIn16	18	acctctataagatcctaacctg	aagggagtgtgactgatatttc	(AC)G(CA)	56	Laucou et al.,2011
VVIp60	1	ggggaataactaaattgaggat	gtatgaatgcggatagtttgtg	(TG)AG(TG)(AG)	56	Laucou et al.,2011
VVS2	11	cagcccgtaaatgtatccatc	aaattcaaaattctaattcaactgg	(GA)	50	Thomas et al.,1993
VVS4	8	ccatcagtgataaaacctaatgcc	cccaccttgcccttagatgtta	(AG)	60	Thomas et al.,1993
VVMD7	7	agagttgcggagaacaggat	cgaaccttcacacgcttgat	(CT)	56	Bowers et al., 1996
VVS5	6	attgatttatcaaacaccttctacat	tagaaagatggaaggaatggtgat	(GT)	55	Thomas et al.,1993
