# Supplementary figures and images for: Morphological and Genetic Clonal Diversity within the ‘Greco Bianco’ Grapevine (Vitis vinifera L.) Variety
Source: Plants (Basel). 2023 Jan 23;12(3):515. doi: 10.3390/plants12030515 (PMC9921137; doi:10.3390/plants12030515)

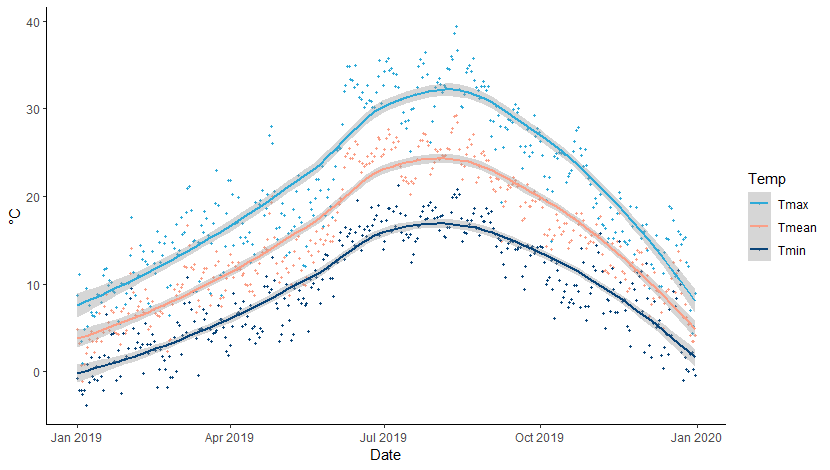

Supplement: Supplementary file 1 [file plants-12-00515-s001.zip › Figure S1. Minimum, mean, and maximum air daily temperature (2 m); each dot represents a daily measurement; lines are smoothed conditional means surrounded by standard error of the means (grey areas).png]
